# Activation of a Cryptic Manumycin-Type Biosynthetic Gene Cluster of *Saccharothrix espanaensis* DSM44229 by Series of Genetic Manipulations

**DOI:** 10.3390/microorganisms9030559

**Published:** 2021-03-08

**Authors:** Dominika Gorniaková, Miroslav Petříček, David Kahoun, Roman Grabic, Tomáš Zelenka, Alica Chroňáková, Kateřina Petříčková

**Affiliations:** 1 Institute of Immunology and Microbiology, 1st Faculty of Medicine, Charles University, Studničkova 7, 12800 Prague, Czech Republic; dominika.gorniakova@lf1.cuni.cz (D.G.); miroslav.petricek@lf1.cuni.cz (M.P.); 2 Faculty of Science, University of South Bohemia, Branišovská 1645/31a, 37005 České Budějovice, Czech Republic; dkahoun@prf.jcu.cz; 3 Faculty of Fisheries and Protection of Waters, University of South Bohemia, Zátiší 728/II, 38925 Vodňany, Czech Republic; rgrabic@frov.jcu.cz; 4 Institute of Microbiology, Academy of Sciences of the Czech Republic, Vídeňská 183, 14220 Prague, Czech Republic; tomas.zelenka.mbu@gmail.com; 5 Institute of Soil Biology, Biology Centre Academy of Sciences of the Czech Republic, Na Sádkách 702/7, 37005 České Budějovice, Czech Republic; alicach@upb.cas.cz

**Keywords:** manumycin, colabomycin, cryptic BGC activation, actinomycetes, *Saccharothrix*, secondary metabolites, immunomodulators, cancerostatics

## Abstract

(1) Background: Manumycins are small actinomycete polyketides with prominent cancerostatic and immunosuppressive activities via inhibition of various eukaryotic enzymes. Their overall activity towards human cells depends on the structural variability of both their polyketide chains, mainly the upper one. In our genetic screening project to find novel producers of anti-inflammatory manumycins, the strain *Saccharothrix espanaensis* DSM44229 was identified as containing a novel manumycin-type biosynthetic gene cluster (BGC). (2) Methods: The biosynthetic genes appeared to be silent under all assayed laboratory conditions. Several techniques were used to activate the BGC, including: (i) heterologous expression in various hosts, (ii) overexpression of putative pathway-specific regulatory genes, and (iii) overexpression of a bottleneck cyclizing aminolevulinate synthase gene in both natural and heterologous producers. (3) Results: Multiple novel manumycin-type compounds were produced at various levels by genetically-modified strains, sharing a tetraene lower chain structure with a colabomycin subgroup of manumycins, but possessing much shorter and saturated upper chains. (4) Conclusions: A cryptic manumycin-type BGC was successfully activated by genetic means to gain production of novel manumycin-type compounds for future comparative activity assays. Heterologously produced compounds were identical to those found after final activation of the BGC in the original strain, proving the intactness of the cloned BGC.

## 1. Introduction

Immensely growing knowledge on the genetics and biochemistry of bacterial secondary metabolism together with the availability of a huge number of bacterial genomic sequences has opened a new space for screening projects to get novel compounds and novel producers via genetic screening and genome mining [1]. This, however, has raised a new challenge for researchers: the identified biosynthetic gene clusters (BGCs) are quite often cryptic and sometimes difficult to activate. Various techniques have been applied to solve the problem, including heterologous expression, ribosome engineering, chemical elicitors application, co-culture approaches, promoter engineering or exchange, and others, reviewed recently by, e.g., Nguyen [2] and Kang [3].

**Figure 1:**
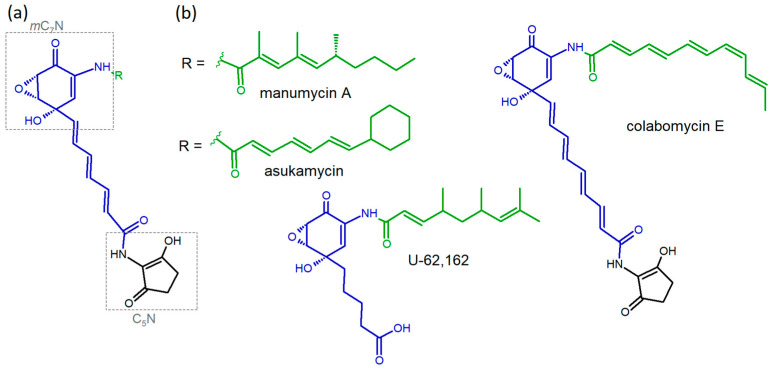
Manumycin-type polyketides. (**a**) General structure. Lower polyketide chains are colored in blue, upper chains in green. (**b**) Examples of variability in both polyketide chains. The lower chain contains 3,4-AHBA as a starter unit prolonged with linear, usually polyene, 4- to 8-carbon chains, but may be saturated in rare cases (U-62,162). The upper chain shows the highest variability affecting length, saturation, branching pattern, and starter unit selection.

Manumycin-type metabolites represent a group of several tens of structurally related small linear polyketides, typically containing two short polyketide chains connected via a central *m*C_7_N cyclic unit derived from 3,4-AHBA (3-amino-4-hydroxybenzoic acid). In the majority of compounds, the lower chain is modified by the attachment of another cyclic unit called C_5_N—(Figure 1) [4,5]. The compounds were first reported as weak anti-Gram-positive antibiotics and certain antiparasitic activities were reported in vitro as well [6]. However, as possible future drug leads, they attracted remarkable attention later on when their potent cancerostatic activity was assessed [7,8] and further studied in numerous works. They specifically inhibit Ras-farnesyl transferase [9] and induce the production of reactive oxygen species (ROS) [10] which both lead to cell proliferation inhibition and apoptosis. Next, they were reported to inhibit inflammatory response via caspase 1 [11] and IKKβ inhibitions [12]. Besides all of these, they inhibit neutral sphingomyelinase and slow down Alzheimer’s disease progression in the mouse model [13]. Manumycin A has been used in the vast majority of all bioactivity studies as it has been commercially available for a long time. According to our comparative studies [14,15] it shows the highest pro-apoptotic features of all manumycin compounds assayed in our laboratory (manumycin A and B, asukamycin and colabomycin E). This qualifies it as a promising anti-cancer agent but complicates its use as a putative anti-inflammatory agent.

The growing incidence of human inflammatory and auto-immune diseases and the general lack of an efficient, low-cost, and low-side effects treatment has opened a new space for small molecules research, in which NFκB pathway components (including the IKK protein kinase) are often mentioned as promising anti-inflammatory drug targets [16]. This motivated us to collect as many producers and compounds from the manumycin group as possible in order to identify those derivatives with strong immunosuppressive features but low apoptosis induction. One of the ways was to perform the genetic screening of natural actinomycete isolates combined with genome scanning of publicly available genomic data to find potential novel producers of manumycin compounds [17]. The screening of about 2000 worldwide natural isolates and several hundred genomes identified a few putative producers, some of which produced already known compounds, whilst some led to the successful identification of new derivatives with promising features, such as colabomycin E [15,18]. Among these, *Saccharothrix espanaensis* DSM44229 was found to carry a typical manumycin-type BGC with genes encoding both cyclic units (C_5_N, *m*C_7_N), components of polyketide synthases for both polyketide chains and subsequent tailoring enzymes, including several variable genes corresponding to the upper chain modifications, and also common regulatory and resistance/transport protein-encoding genes. However, primary attempts to identify even traces of a manumycin-type compound in the strain culture extracts failed under all culture conditions used.

The major aim of this work was to assess whether the genetic information of the manumycin-type BGC in *Saccharothrix espanaensis* DSM44229 is not damaged but just silenced to zero activity under laboratory conditions by unknown regulatory circuits of the native producer. This required, as the first step, a thorough analysis of the BGC genetic information, including surrounding regions. Next, it was necessary to assess its activity in vivo in suitable heterologous producers. Finally, we activated it back in the original organism and compared the chemical structures detected in natural and heterologous producers. This allowed us to assess the possible effects of the heterologous producer genetic background on the spectrum of produced compounds compared to the native producer.

## 2. Materials and Methods

### 2.1. Saccharothrix espanaensis DSM44229 Strain Acquisition

The strain was purchased from the German Collection of Microorganisms and Cell Cultures (DSMZ) after it was been identified as carrying a cyclizing aminolevulinate synthase (cALAS) gene homologue closely related to cALAS genes from manumycin-type BGCs [17]. The associated genome is publically available (NCBI Reference Sequence: NC_019673.1).

### 2.2. Cultivation Media

*Saccharothrix espanaensis* pilot fermentations were performed in SN, STX, SSY, SSM, and GYM media. SN medium (pH 7.2) contained (per liter): neutralized bacteriological peptone (Oxoid, Basingstoke, UK), 5 g; casein hydrolysate, 1 g; KH_2_PO_4_, 0.25 g; MgSO_4_·7H_2_O, 0.5 g; CaCO_3_, 2 g; NaCl, 2 g; glycerol, 30 g; and 10 mL of 100× salts stock (per liter: (NH_4_)_6_Mo_7_O_24_·4H_2_O, 0.5 g; FeSO_4_·7H_2_O, 5 g; CuSO_4_·5H_2_O, 0.5 g; ZnSO_4_·7H_2_O, 0.5 g; MnCl_2_·4H_2_O, 1 g). STX medium, pH 7.2, contained, per liter: molasse, 10 g; CaCO_3_, 5 g; NaCl, 2 g; and glucose, 20 g and was dissolved in soya flour extract (40 g of soya flour boiled in dH_2_O, 1 L, for 20 min and filtered). SSM medium was composed of (per liter, pH 7.2): glucose, 10 g; (NH_4_)_2_SO_4_, 2 g; NaCl, 2 g; KH_2_PO_4_, 0.5 g; K_2_HPO_4_, 1 g; MgSO_4_·7H_2_O, 0.2 g; CaCO_3_, 5 g; and yeast extract (Oxoid, Basingstoke, UK), 2 g. SSY medium (pH 7.2) contained, per liter: soya flour, 20 g; saccharose, 10 g; starch, 5 g; yeast extract (Oxoid, Basingstoke, UK), 2 g; neutralized bacteriological peptone (Oxoid, Basingstoke, UK), 2 g; NaCl, 2 g; CaCO_3_, 1 g; MgSO_4_·7H_2_O, 0.5 g; and KH_2_PO_4_, 0.5 g. GYM standard medium contained, per liter: glucose, 4 g (or glycerol, 10 g, alternatively); yeast extract (Oxoid, Basingstoke, UK), 4 g; malt extract (Oxoid, Basingstoke, UK), 10 g; NZ amine (type A, Wako), 1 g; NaCl, 2 g; and 3 mL of OB salts solution stock (containing, per liter: CuSO_4_·5H_2_O, 1.66 g; FeSO_4_·7H_2_O, 2.5 g; MnSO_4_·5H_2_O, 1.2 g; CaCl_2_·2H_2_O, 5 g; and ZnSO_4_·7H_2_O, 3 g).

Streptomycetes were cultured in YEME and R2YE for protoplast transformation [19] and GYM medium for fermentation, supplemented with apramycin (100 μg/mL) and/or thiostrepton (30 μg/mL) when needed at 28 °C.

*Escherichia coli* was cultivated in standard liquid or solid LB media supplemented with antibiotics when needed [20] at 37 °C. For conjugation, LB medium was supplemented with 0.1% glucose [21].

### 2.3. Fermentation

Standard fermentation was performed in 0.5 L baffled Erlenmeyer flasks (80 mL of media per flask), 28 °C, 200 rpm. Seed cultures were inoculated from five-day-old sporulated agar culture and cultured for two days. The seed cultures were then transferred into a fresh medium (1/10 of the final volume) and cultured for another three days under the same conditions if not stated differently.

### 2.4. Metabolite Extraction

Metabolic extracts were prepared by organic solvent extraction of both post-culture media and mycelia with ethylacetate/acetone as follows: the mycelium was first extracted with acetone (0.5 volume of original culture) for 30 min, 4 °C, 200 rpm reciprocal shaking. The cell pellet was removed by centrifugation and the acetone was evaporated using a rotary evaporator at 90 mbar pressure and 37 °C bath temperature. The residuum was then re-extracted with three volumes of ethylacetate as below. The post-culture medium was first supplemented with NaCl to 5 M concentration and subsequently extracted with one-third of the culture volume of ethylacetate under the same conditions. The phases were separated by centrifugation and the ethylacetate fraction was combined with the ethylacetate extract of the mycelium and dried in the vacuum evaporator. The extract was dissolved in 1/1000 of the original culture volume in chloroform and stored under a nitrogen atmosphere at −80 °C. For the HPLC analysis, 5 μL of the extract were mixed with an equal volume of acetonitrile (ACN) and applied.

Purification of metabolites for NMR analysis was undertaken with two independent procedures. The first one used standard column chromatography as described by Petrickova [18]. The second deployed FLASH chromatography using a VersaPak Silica Cartridge Column from Supelco (Bellefonte, PA, USA), with a linear gradient of chloroform/methanol ranging from 99:1 to 90:10 (*v*/*v*). The fractions were collected and analyzed by UHPLC-MS.

### 2.5. Analytical Techniques

For fast screening, TLC using aluminum sheets with Silica gel containing F254 indicator (Merck, Darmstadt, Germany) were used; benzene/acetone, 3:2 (*v*/*v*), was used as a mobile phase. The plates were then air-dried and photographed under UV illumination or used for the in situ antibiotic activity assay using *Bacillus subtilis* subsp. *spizizenii (ATCC^®^ 6633™)*.

UHPLC was used for pilot monitoring of the compounds production. UHPLC-DAD-ToF-MS analyses were carried out with a Waters Acquity UPLC System (Waters, Manchester, UK). The Waters LCT Premier XE orthogonal accelerated time-of-flight mass spectrometer (Waters MS, Manchester, UK) with an electrospray interface was operated in both positive and negative ion modes. Full scan spectra from *m*/*z* 100 to 1200 were acquired with a scan time of 0.1 s and 0.01 s interscan delay. The fragmentation by means of in-source collision induced dissociation (CID) was achieved with the Aperture I value set to 50, 75, and 100 V. Mass Lynx V4.1 software was used for data processing. Analyses were performed on an Acquity UPLC BEH C18 column (502.1 mm i.d., 1.7 mm) with a mobile phase flow rate of 0.4 mL·min^−1^, column temperature of 25 °C, and injection volume of 1 mL. The mobile phase consisted of (A) formic acid/water (0.1:99.9, *v*/*v*), and (B) ACN, with the gradient elution program (min/% A) as follows: 0.0/90.0, 12.0/40.0, 15.0/20.0, 16.0/20.0, followed by a 2.0 min wash step with 100% B. After acquisition, the specific (MH) ions were extracted with a 0.02 Da extraction mass window. For verification of compound identities, the parameters set for Elemental Composition editor were: CHNO algorithm; mass measurement error tolerance, 5 mDa; i-FIT (norm) error, 5. Fragmentation by CID was employed for verification of generated fragment ions with a mass measurement error tolerance of 10 mDa set in Mass Fragment software.

High-resolution HPLC-MS/MS (LC-HRMS) analysis was used for detailed comparative analyses of production patterns. Ten microliters of extract were injected by autosampler (PAL-RXi, CTC Analytics AG, Zwingen, Switzerland) onto a HypersilGoldTM analytical column (50 mm length × 2.1 mm ID and 5 µm particle size (ThermoScientific, Waltham, MA, USA) preceded with the same stationary phase precolumn (10 mm length × 2.1 mm ID and 5 µm particle size). Analytes were separated using the following gradients of acetonitrile in water (both acidified with 0.1% formic acid): 100% water for 1 min at flow rate 350 µL·min^−1^, linear gradient to 25% of ACN in 4 min at flow rate of 350 µL·min^−1^, 60% ACN in 8 min, and then to 100% ACN in 10 min, followed by 2 min 100 ACN isocratically, all at 450 µL·min^−1^, then 100% water in 12.01 min and 3 min equilibration at initial conditions.

Hybrid orbital trap HRMS (QExactive HF, Thermo Scientific, Waltham, MA, USA) was operated in electrospray ionization full-scan high-resolution mode combined with data-independent analysis mode. One LC-MS method was run in positive and another in negative ionization mode. Electrospray conditions were equal for both positive and negative modes (42 AU-arbitrary unit sheath gas, 10 AU auxiliary gas, vaporizer 250 °C, and capillary 325 °C) except for ionization voltage, which was 3500 V for the positive and 2800 V for the negative mode. Full scan spectra were acquired with the following settings: mass range 100 to 1300 *m*/*z*, resolution 60,000 FWHM, AGC target 3.106, and ion time 50 ms. DIA experiment was run with a 100 *m*/*z* isolation window for m/z 150, 250, 350, 450, 550, and 650; stepped collision energy of 15%, 35%, and 60% of the normalized collision energy (NCD); AGC target 1.106; and ion time 30 ms. Data were processed with Xcalibur and MassFrontier 7.1 software (Thermo Scientific, Waltham, MA, USA).

NMR spectra were recorded with a Bruker Avance III 600 (600.23 MHz for 1H, 150.94 MHz for 13C) and (D7) DMF (ARMAR AG, Dottingen, Switzerland) at 30 °C. Residual signals of solvent were used as internal standards (dH = 2.743 ppm, dC = 30.11 ppm). The NMR experiments 1H NMR, 13C NMR, gCOSY, J-resolved, gHSQC, gHMBC, and 1D TOCSY were performed with the manufacturer’s software. The 1H NMR and 13C NMR spectra were zero-filled to fourfold data points and multiplied by window function before Fourier transformation. A two-parameter, double-exponential Lorentz–Gauss function was applied for 1H to improve resolution, and line broadening (1 Hz) was applied to get better 13C signal-to-noise ratios. Chemical shifts are given on the d scale with digital resolution justifying the reported values to three (dH) or two (dC) decimal places.

### 2.6. Cosmid Library Preparation and Analysis

The genomic DNA of DSM44229 was isolated by the large-scale technique described by Kieser et al. [22]. The 35–40 kbp fragments were generated using partial cleavage by *Sau*3AI restriction endonuclease and ligated with *Hpa*I-*Bam*HI generated arms of the pOJ446 cosmid vector using a standard protocol [22]. The recombinant cosmid clones were packaged using a Gigapack III Gold Packaging Kit (Agilent) and the phage particles used for the *E. coli* VCS257 cells infection following the manufacturer protocol. The infected cells were plated on LB agar supplemented with apramycin (100 μg/mL). Cosmids carrying the manumycin-type BGC were identified by colony hybridization using a DIG-labeled fragment (DIG DNA Labelling Kit, Roche, Indianopolis, IN, USA) of the cALAS-encoding gene—generated by PCR using primers originally designed for the *asuD2* (*hemA*) gene homologues screening in natural actinomycete isolates, HEMA1 and HEMA3 [17]—as a probe; genomic DNA of DSM44229 was used as a template. As a secondary probe, the *espA1* fragment, specific for the *m*C_7_N unit genetic information, was generated by PCR using primers designed for screening purposes (AHBASF: 5′-AAGATCCSYTSGAGATCGTSGTSGC-3′ and AHBASR: 5′-SGGCATGTACGGSARSGGGTGSGTCTC-3′). The cosmid DNA from the double-positive clones was extracted with a High Pure Plasmid Isolation Kit (Roche), cleaved with *Mlu*I and *Kpn*I restriction endonuclease, and the fragment pattern was compared to that predicted from the DSM44229 genomic sequence of the BGC locus.

### 2.7. Construction of Strains for Heterologous Expression

The cosmid clones carrying the entire BGC were transformed in the *E. coli* ET12567/pUZ8002 strain to carry out conjugation to recipient streptomycete strains specialized for the secondary metabolite heterologous production by the technique developed for the REDIRECT mutagenesis system [21]. *Streptomyces lividans* K4-114 [23] and *S. coelicolor* M512 [24] were used as the recipients.

### 2.8. Overexpression Constructs

cALAS-encoding *asuD2* gene from the asukamycin producers *S. nodosus* ssp. *asukaensis* was fused to the ermE* constitutive promoter and cloned in two streptomycete conjugative vectors: replicative pKC1218 [22] and integrative pMS17 [25]. The resulting constructs were named pALS4K and pALS4KON, respectively. The pALS4K plasmid was previously shown to stimulate the manumycin-type metabolite production in various producers (unpublished). The plasmid presence was checked using PCR with ERMF2 (5′-ATGCTGTTGTGGGCACAATC-3′) and HEM3 primers.

The regulatory genes *espR1* and *espR2* were amplified individually by PCR using the following primers: ESPR1F (5′-AGGATCCAGGGTCGACCGACGTGCT-3′) and ESPR1R (5′-CGCATGCCATTTCCCCAGCTTACCC-3′) or ESPR2F (5′-CGCATGCCGGGTCTGCGACATCTG-3′) and ESPR2R (5′-CAAGCTTAATCGCGTTCGGGCTCCT-3′), respectively. Next, both genes were amplified in a single fragment using ESPR1F and ESPR2R. Genomic DNA of DSM44229 was used as the template. The fragments were fused to the ermE* promoter and cloned in the pIJ487 plasmid vector [22]. The resulting plasmid clones were named pESPR1, pESPR2, and pESPR1+2, respectively. The plasmids were used to overexpress the regulatory genes in *S. lividans* K4-114-derived producers.

The ermE*-*espR1* fragment was cloned in pKC1218 and pMS17 vectors, too. The resulting constructs, pESPR1K and pESPR1KON, were used to overexpress the regulatory gene in the parent DSM44229 strain. The plasmid presence was checked using PCR with ERMF2 and ESPR1R primers.

## 3. Results

### 3.1. Manumycin-Type BGC Identification in Saccharothrix espanaensis DSM44229

The strain was identified as carrying the cALAS gene homologue in our previous actinomycete natural isolates screening and genome scanning project [17]. The organism, isolated from the soil sample in Puerto Llano, Spain, has been previously reported to produce other secondary metabolites, heptaglycoside saccharomicins [26], and its large genome (9.3 Mbp) was sequenced in 2012 [27].

**Figure 2:**
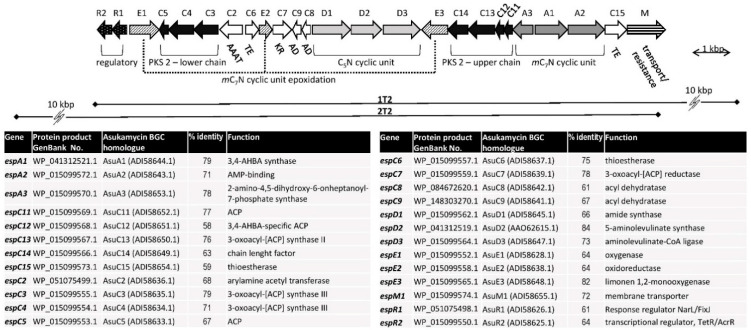
Manumycin-type gene cluster of *Saccharothrix espanaensis* DSM44229. The genes were assigned by homology to the genes of the asukamycin biosynthetic gene cluster (BGC) in *Streptomyces nodosus* ssp. *asukaensis* ATCC 29757, GenBank: *GQ926890.1* [28]. The gene functions and homology to asukamycin biosynthetic genes are listed in the table below. 1T2 and 2T2 cosmid clone inserts extents are indicated.

The genome contains three copies of cALAS gene homologues, identified by BN6_21290, BN6_48530, and BN6_50240 locus tags in the genomic DNA GeneBank record. In all three cases they are accompanied by the other two genes needed to form and attach the C_5_N unit to metabolite core structures. DSM44229 is, to our knowledge, the only organism carrying three copies of the C_5_N-encoding locus in its genome, though the functionality of the last two is unclear. The BN6_21290 gene (*espD2*) is located within a typical manumycin-type BGC, in a typical C_5_N-encoding operon together with homologues of C_5_N-specific amide synthase (espD1) and aminolevulinate-CoA ligase (*espD3*) genes. The BGC contains all the genes essential for the biosynthesis of core structures of manumycins (Figure 2). For comparison with previously characterized manumycin-type BGCs, see Figure S1. It should be noted that, compared to the other related BGCs, it does not contain any unique, upper chain variability-related genes (Figure S1). Based on the cluster comparison, we can suggest that the upper chain may originate from general PKS precursors, considering both starter and extender unit selection, and undergo no further polyketide chain tailoring steps. The putative protein products of PKS-encoding *espC3*–5, acyl dehydratase *espC8,9*, and chain-attachment (arylamine acyl transferase) *espC2* genes show high homology to the proteins responsible for the biosynthesis of linear upper chains of asukamycin and colabomycin (between 67–79% identical amino acid residues in *espC2–C5*, slightly less in the acyl dehydratases) (see Figure 2 and Figure S2).

### 3.2. Basal Activation of the BGC by Heterologous Expression

In pilot fermentation experiments, the DSM44229 strain was cultivated in SN, STX, GYM media, which we regularly use for fermentation of other producers of manumycin-type compounds. Standard ethylacetate/acetone extraction was applied, as commonly used for manumycin-type compounds [18]. The extracts were subjected to UHPLC-MS/MS analysis, for which no traces of manumycin-type compounds were identified. Typical retention times, molecular weight, UV absorption spectrum, and presence of MS/MS fragments (ESI^−^) specific for manumycins, derived from the conserved part of the lower chain with the C_5_N unit attached, were considered as markers (112.0393, 138.0187, 178.0501) [18]. Next, the fermentation was performed using selected media for pseudonocardias, SSY, and SSM. Again, no traces of any manumycin-related compounds were detected in the extracts by UHPLC. This indicated that the BGC is perhaps silent under standard laboratory conditions in the wild-type strain.

In order to verify the intactness of the BGC, a genomic cosmid library of the strain was prepared using pOJ446 cosmid (*E. coli*—*Streptomyces* shuttle cosmid vector allowing low-copy number replication in streptomycetes). Fourteen *E. coli* clones carrying the BGC were detected by colony hybridization with the *espD2* cALAS gene-specific probe, of which 12 clones hybridized with the secondary *espA1*-specific probe as well. By restriction endonuclease mapping, the clones were aligned to the known DSM44229 genomic sequence of the BGC (not shown). Two clones, 1T2 and 2T2, were chosen to be used in heterologous expression experiments. Both clones contained the entire putative BGC locus with approximately 10 kb extensions down- or upstream, respectively (Figure 2).

**Figure 3:**
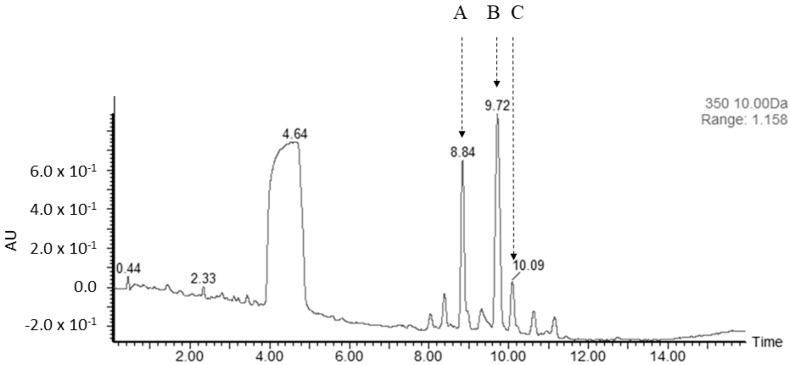
Activation of the manumycin-type BGC by the heterologous expression, UHPLC record. Three novel metabolites were produced in K4-114 × 1T2 and detected at λ = 350 nm: A: ES-: 507.2173, C_28_H_32_N_2_O_7_, B: ES-: 521.2343, C_29_H_34_N_2_O_7_, C: ES-: 533.2308, C_30_H_34_N_2_O_7_.

Both cosmid clones were introduced in the heterologous host cells, *S. lividans* K4-114 and *S. coelicolor* M512, by conjugation and their presence was verified using PCR, detecting *asuD1* and *asuA1* homologues (the same primers as used for the probe preparation). The verified strains were cultivated in GYM medium under standard fermentation conditions (two days of preculture, three days of fermentation culture); secondary metabolites were extracted and analyzed both with TLC (Figure S3a,b: lanes 1 and 2) and UHPLC-MS (Figure 3). The presence of any of the two cosmid clones induced the production of three novel manumycin-type compounds in small amounts. The best production, based on the relevant peak area, appeared in K4-114 × 1T2 (Figure 3 and Figure S3): the production level was approximately 20 times higher than in the case of K4-114 × 2T2. Comparably lower production levels were found in M512-derived producers (not shown).

**Figure 4:**
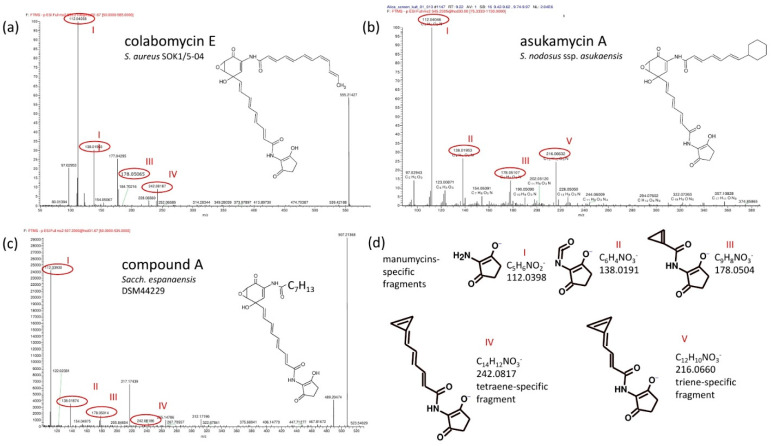
Comparison of ESI^−^ LC-MS/MS spectra of tetraene colabomycin E (**a**), triene asukamycin A (**b**), and compound A (**c**) The corresponding fragment structures (**d**) were predicted by Mass Frontier 7.1 software (Thermo Scientific) based on the exact molecular mass. Typical fragments shared by all manumycins containing the C_5_N unit are shown (I, II, III) together with trienic (V) and tetraenic (IV) lower chain-specific fragments.

The detailed ESI^−^ MS/MS fragmentation analysis was performed using LC-HRMS. The fragments generated from the compound A are shown in Figure 4 in comparison to fragmentation spectra of the triene-type (asukamycin A) and tetraene-type (colabomycin E) manumycins. All three compounds shared the structure of the lower polyketide chain with colabomycins—i.e., tetraene unsaturated lower polyketide chains—but possessed shorter and more saturated upper chains (Figure 4) compared to the most structurally related compounds colabomycin A [29] and E [18]. Almost identical spectra as those for compound A were acquired for compounds B and C, too (not shown). The results suggest that all the three novel compounds (A, B, C) share a tetraene lower chain structure with the upper chains composed of C_7_H_13_, C_8_H_15_, and C_9_H_15_, respectively. Metabolites A and B are major products, C is a minor congener.

### 3.3. Effect of Pathway-Specific Regulators on the Production of Manumycin-Type Compounds in the Heterologous Producer

In order to support the production of the K4-114 × 1T2 heterologous producer we cloned both putative pathway-specific regulatory genes, *espR1* and *espR2*, under the control of a strong constitutive streptomycete ermE* promoter, both for each independently and combined in a single construct, using a multicopy streptomycete pIJ487 vector. The genes are homologous to *asuR1* and *asuR2* asukamycin BGC regulatory genes, where *asuR1* codes for the cluster key positive regulator [30]. The resulting plasmids were named pESPR1, pESPR2, pESPR1+2, respectively. The plasmids together with the empty vector control were used for K4-114 × 1T2 protoplast transformation with subsequent apr^R^ thio^R^ selection. pESPR1 transconjugants turned notably yellow compared to the control strain and other recombinant strains (Figure S3c). Based on our previous experience, this was an indication of promoted production of manumycin-type pathway intermediates.

**Figure 5:**
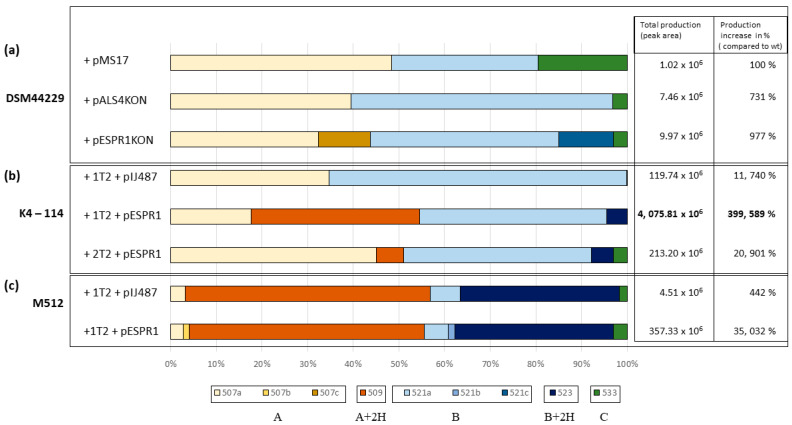
LC-HRMS analysis of the production of manumycin-type compounds encoded by the DSM44229 BGC in various producer strains (ESI^−^). The list of strains is provided in the left column, the total amounts of manumycin-type compounds produced (relative amounts reflected by peak areas) and relative production levels calculated as percentages of that in DSM44229 × pMS17 are shown in the right columns. The best producer strain is shown in bold. Composition and ratio of the compounds produced are indicated for each strain in color: compound A in three isomers sharing the same MS/MS fragmentation pattern, but showing different retention times (507a: 7.57 min, 507b: 7.03 min, 507c: 5.72 min); type II form of compound A (A+2H)—two isomers double peak (509: 7.01–7.21 min); compound B in three isomers (521a: 7.94 min, 521b: 7.38 min, 521c: 6.04 min); type II form of compound B (B + 2H)—two isomers double peak (523: 7.36–7.55 min), and the minor congener compound C (533: 8.10 min). (**a**) *Saccharothrix espanaensis* DSM44229-derived strains, (**b**) *Streptomyces lividans* K4-114-derived strains, (**c**) *Streptomyces coelicolor* M512-derived strains. Amounts of the extract loaded were equal to 4 mL of the original post-fermentation culture volumes.

The extracts of all four strains, together with K4-114 and K4-114 × 1T2, were subjected first to TLC (Figure S3) and then to LC-HRMS analysis, and production levels were compared. The majority of the strain extracts did not show any remarkable increase in production levels, except for K4-114 × 1T2 and, even higher, K4-114 × 1T2 × pESPR1. The total production was almost 40 times higher in K4-114 × 1T2 × pESPR1 compared to the K4-114 × 1T2 × pIJ487 control, as measured by the relevant peak area sum in LC-MS (Figure 5b). This suggests that *espR1* serves as a pathway-specific positive regulator. The role of *espR2* seems to be rather negative, as production levels of K4-114 × 1T2 × pESPR2 and K4-114 × 1T2 × pESPR1+2 were as low as in the control strain.

In the last experiment we used a different recipient strain, *S. coelicolor* M512. The total production of manumycin-related compounds was about one order lower than in the corresponding K4-114-derived strain. Interestingly, hydrogenated forms of the two main products, A and B, were produced as major products in these strains (Figure 5c). These, based on their mobility in the HPLC system, molecular mass, and fragmentation pattern, represent type II variants of A and B (Figure S4). The presence of the type II forms is common in all manumycin-type compound producers, but it is not clear whether the process of type I to type II conversion is caused by the instability of the type I compounds or relies on the genetic/enzymatic background of a particular producer [28].

### 3.4. Activation of the BGC in the Parent Strain

In order to elucidate the effects of particular genes of the cluster overexpression directly in the parent strain DSM44229, we prepared two constructs allowing overexpression of the *espR1* regulatory gene and *asuD2* under the control of the ermE* promoter. The activating effect of *espR1* has already been shown in the heterologous producers. The overexpression of the *asuD2* gene, coding for the cALAS of the asukamycin pathway, was previously shown to promote the production of the C_5_N-containing metabolites in various producers in our laboratory. The enzyme activity probably represents one of the pathway rate-limiting steps and increasing the gene expression may thus promote the compound formation rates. In this case, the promoter-gene fusion cassettes had to be cloned in the conjugative vectors as all our attempts to transfer plasmid constructs by other techniques in DSM44229 failed (PEG-mediated protoplast transformation, electroporation). First, the replicative low-copy pKC1218 vector was used, resulting in pALS4K and pESPR1K; however, no apr^R^ transconjugants were found in any of multiple experiments using either spore or mycelia recipient DSM44229 cells. To exclude the failure of pKC1218 replication in the DSM44229 cells, we also cloned the cassettes in the conjugative pMS17 vector, which does not replicate in the recipient, but integrates into the host chromosome via ΦC31 *attC* site. This approach in both constructs (pALS4KON, pESPR1KON) led to the acquisition of several thousand transconjugants per plate. The genomic DNA of a few selected clones was isolated using a Wizard DNA Isolation Kit (Promega, Madison, WI, USA) and the presence of *asuD2* (pALS4KON) or *espR1* (pESPR1KON) fused with ermE* was verified by PCR using ERMF2-HEM3 and ERMF2-ESPR1R primer pairs with fragment sizes of 782 bp and 902 bp, respectively.

The verified clones (DSM44229 × pALS4KON and DSM44229 × pESPR1KON) were subjected to fermentation in GYM medium and subsequent extraction under the standard conditions. DSM44229 carrying an empty pMS17 vector was used as a wild-type control. LC-HRMS, providing higher sensitivity than the UHPLC used in initial pilot fermentations, was used for the analysis. In all three strains, small amounts of the three major compounds A, B, and C were produced. Overexpression of heterologous, asukamycin BGC-related *asuD2* increased production of manumycin-type compounds by approximately seven times and overexpression of *espR1* by almost ten times, compared to the DSM44229 × pMS17 control (Figure 5a).

### 3.5. Structure Studies of the Novel Compounds

**Figure 6:**
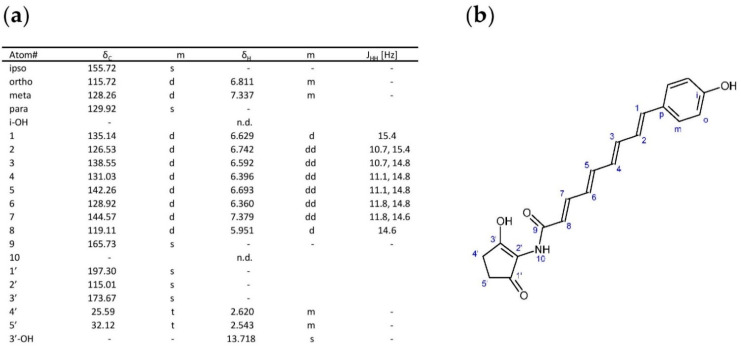
Pathway intermediate structure predicted by NMR. Measured for ^1^H and ^13^C at 700.13 MHz for ^1^H and at 176.05 MHz for ^13^C in CDCl_3_ at 20 °C. (**a**) NMR data summary, (**b**) structure predicted.

The 3L-fermentation culture of K4-114 × 1T2 × pESPR1 was used to purify the compounds and assess their structure by NMR. The purification protocol followed the one described for colabomycin E [18]. However, the majority of the main compounds tended to degrade during the purification chromatographic steps. Finally, we succeeded in analyzing only the structure of one of the pathway intermediates without the upper polyketide chain (Figure 6). The intermediate structure proofs the tetraene, colabomycin-type structure of the lower polyketide chain.

The structure of the upper chain can only be deduced from the LC-MS/MS data combined with the lower chain NMR-based data. Some predictions can also be made based on the analysis of the genetic information responsible for the upper chain structure (Figures S1 and S2). Comparing the relevant genetic information to BGCs encoding other manumycin compounds (Figures S1 and S2), it seems that linear structures of the upper chain are more probable; however, this has to be proven by NMR in the future.

## 4. Discussion

Like many other secondary metabolites, manumycins possess numerous bioactivities based mostly on the enzyme inhibition mechanism. Up to now, they have mostly been studied for their cancerostatic activity, with over 350 studies published on the topic so far. The compounds, however, show comparably attractive immunosuppressive features, mainly targeting inflammatory processes [15,31]. Moreover, our pilot data with human peripheral blood-originated buffy coats suggest their suppressive influence on T-cell cytokine production as well (unpublished). The spectrum of the reported activities suggests that this type of compound may, in the future, serve as a novel drug lead for the treatment of chronic inflammatory diseases—e.g., inflammatory bowel diseases, rheumatic arthritis, etc.—but also for suppression of various severe immunopathologies associated with infectious diseases (COVID-19, hepatitides, etc.). Based on our previous work [15], it seems that the upper polyketide chain represents a key structure of the molecules for determining whether pro-apoptotic, cancerostatic activities prevail over the immunosuppressive ones, which is the case for the commercially available manumycins A and B. On the other hand, compounds isolated and assayed in our laboratory (asukamycin, colabomycin E, and others) showed less prominent pro-apoptotic features [15]. It has already been proven that one of the processes crucial in the inhibition of cell proliferation is the inhibition of Ras-specific farnesyl transferase [9]. The responsible molecular mechanism seems to originate from the competitive inhibition of farnesyl-PP binding by the manumycin A upper polyketide chain [32]. Furthermore, manumycins induce the production of reactive oxygen species, promoting apoptosis as well. The exact mechanism is not clear in this case but may include the IL-1β signaling pathway, redox impairment, and mitochondrial membrane damage and the above-mentioned Ras pathway interference [33,34]. Variation of the upper chain structure may thus lead to the substantial reduction of pro-apoptotic features, which was the main reason to search for novel manumycin class compounds in our project.

*Saccharothrix espanaensis* DSM44229 was found as one of the strains carrying a typical manumycin-type gene cluster in its genome during our cALAS-encoding gene screening project [17]. It is also the only organism carrying two more unrelated BGCs containing triplets of the C_5_N biosynthesis-encoding genes elsewhere in the genome [17]. Compared to the first cALAS-positive strain found in the screening, *Streptomyces aureus* SOK1/5-04 (the novel colabomycin E producer, [17]), the BGC was silent in this organism under all assayed laboratory conditions. Compared to other manumycin-type BGCs cloned and analyzed in our laboratory so far—asukamycin [28], colabomycin E [17], manumycin, and U-62,162 (preliminary unpublished data)—it was the smallest, containing only the core biosynthetic genes without any specific genes encoding unusual chain-tailoring enzymes or enzymes needed for atypical upper-chain starter-chain precursor biosynthesis as seen in other clusters (Figure S1). The BGC may have been affected by genome rearrangement events as it is surrounded by IS1182 family transposase (WP_041316446.1) genes, as well as those of another transposase (WP_015099577.1), on both sides.

Heterologous expression of the BGC proved that the BGC contains sufficient and functional genetic information for the production of three previously unknown manumycin-type metabolites, though the production levels remained at low levels in both of the two used heterologous producers. However, subsequent experiments with two putative pathway-specific regulatory genes showed that the production can be substantially elevated by overexpression of one of them, *espR1*. *EspR1* is homologous to the key positive regulatory gene of the asukamycin BGC, as reviewed previously [30]. The same compounds as found in the heterologous producer, judged by high-resolution LC-MS and MS/MS fragmentation spectra, could also be traced back, on the edge of detection limits, to the wt DSM44229 strain by specific LC-MS/MS fragments-targeted analysis of the extract. In K4-114- and DSM44229-derived strains, the compounds A and B prevailed as two major products; C was produced as a minor congener in most of the strains except for when DSM44229 × pMS17 used as a wild-type control. However, the amounts of all the compounds produced were extremely low in the wild strain. Interestingly, the heterologous expression of the BGC in *S. coelicolor* M512 remarkably shifted the production towards hydrogenated variants of the two major products. Based on the previous knowledge of the asukamycins production reported in detail in *S. nodosus* ssp. *asukaensis*, and observed higher polarity of the hydrogenated compounds compared to the corresponding A and B compounds, we suppose that these represent so-called type II manumycins with the C5, C6 epoxy group reduced to a 5-hydroxyethylene. These are commonly found in various producers of manumycin-type compounds [28]. It is not clear whether the conversion is an enzymatic or nonenzymatic process. Our data suggest strain-dependency of the conversion, which may support unspecific enzymatic conversion influenced by the different genetic and metabolic backgrounds of the producers.

We failed in the final structure characterization step, purification of the final products for NMR structural assessments from the K4-114 × 1T2 × pESPR1 fermentation culture. One of the reasons was the substantial instability of the producer strain and instability of the compounds during purification steps. Finally, we succeeded in purifying at least one of the intermediates accumulated in the strain extract, the lower chain with both cyclic units attached. Its NMR structural data proved the interpretation of previous LC-MS/MS data and the suggested tetraene structure of the lower chain. The structure of the upper chains is, unfortunately, still unclear. However, in the phylogenies of the upper chain biosynthesis- and attachment-encoding enzymes, namely PKS subunits (C3–C5) and chain-attaching arylamine acyl transferase (C2), the DSM44229-encoded proteins clearly cluster with those from producers of other manumycins with linear upper polyketide chains: asukamycin and colabomycin (Figure S2). Next, it was found that the DSM44229 cluster does not encode any extra genes which could possibly be associated with unusual chain tailoring or biosynthesis of atypical chain precursors (Figure S1). Based on these findings it is probable that the structure of the upper chain is simple and linear, which should be proven by NMR in the future. For this purpose, the construction of a production strain for more stable BGC expression is needed. As manumycins also share weak antibacterial features, with unknown molecular mechanisms of action, we cannot exclude toxicity of the products for the heterologous host streptomycetes due to improperly working resistance mechanisms. The selection of the heterologous host may thus be crucial. Also, switching from a self-replicating cosmid vector to an integrative one and direct promoter manipulation in the regulatory genes there may help to stabilize the producer strain. Unfortunately, direct manipulations in *Saccharothrix* are less convenient due to the incompatibility of streptomycete-derived common replicons—which has already been reported before in other *Pseudonocardiaceae*, e.g., in *Actinoplanes* [35]—and the lack of specific genetic tools.

## Data Availability

Not applicable.

## Supplementary Materials

The following are available online at https://www.mdpi.com/2076-2607/9/3/559/s1, Figure S1: Comparison of manumycin-type BGCs. Figure S2: Phylogeny of the upper polyketide chain–specific shared enzymes in manumycin-type BGCs of asukamycin (asu), manumycin (man), colabomycin E (col), U-62,162 (ver), and DSM44229 (esp) [36]. Figure S3: Analysis of heterologous production, including the effect of *espR1* and *espR2* overexpression on the production of manumycin-type compounds. Figure S4: MS/MS fragmentation spectra of type I (MW 508) and type 2 (MW 510) manumycin metabolites encoded by a DSM44229 manumycin-type BGC (ESI^−^).
