# Supplementary material for: Activation of a Cryptic Manumycin-Type Biosynthetic Gene Cluster of Saccharothrix espanaensis DSM44229 by Series of Genetic Manipulations
Source: Microorganisms. 2021 Mar 8;9(3):559. doi: 10.3390/microorganisms9030559 (PMC8000086; doi:10.3390/microorganisms9030559)
Supplement: Supplementary file 1 [file microorganisms-09-00559-s001.zip › Supplementary Figures.pptx]

## Slide 1
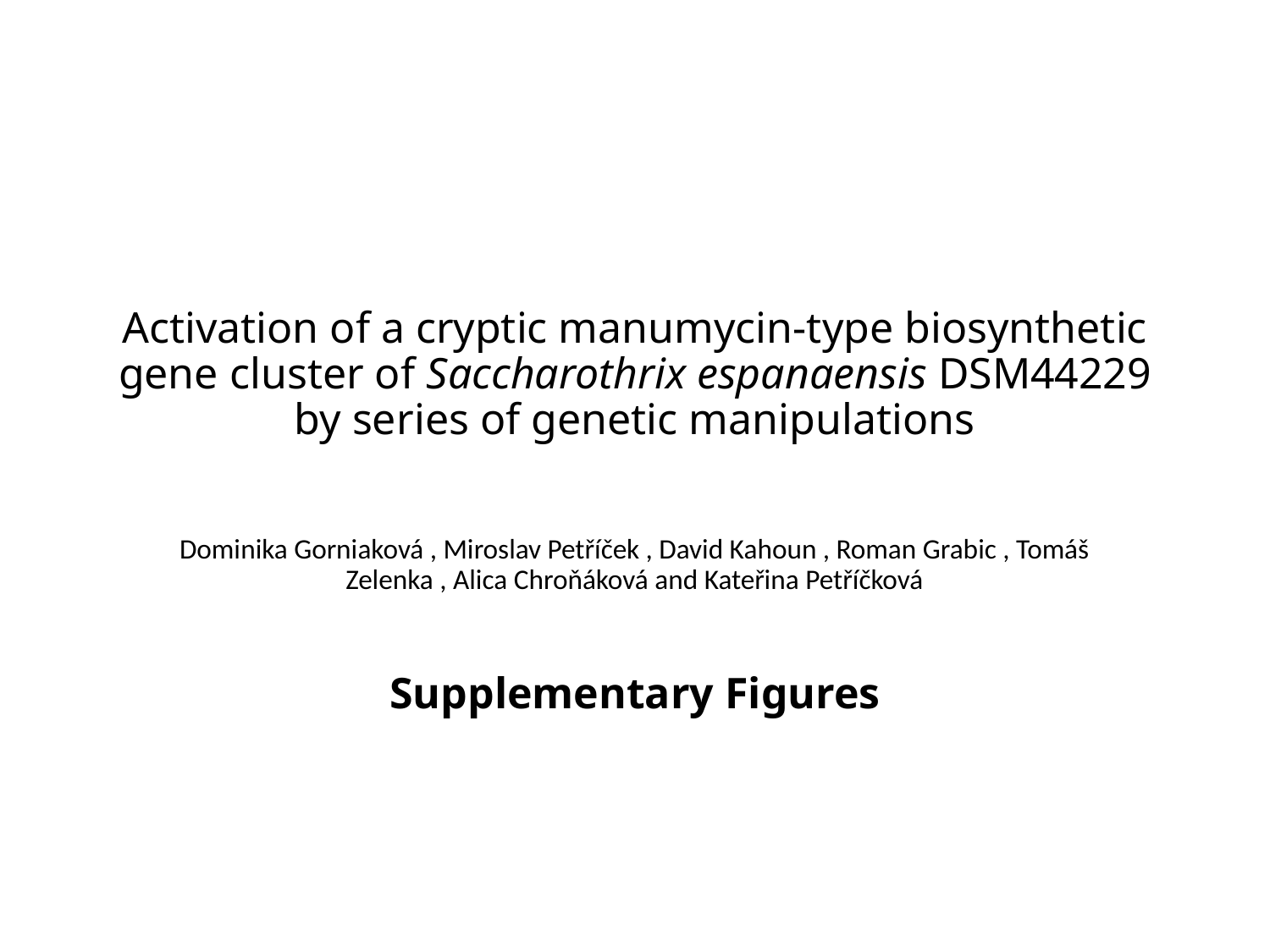

# Activation of a cryptic manumycin-type biosynthetic gene cluster of Saccharothrix espanaensis DSM44229 by series of genetic manipulations Supplementary Figures
Dominika Gorniaková , Miroslav Petříček , David Kahoun , Roman Grabic , Tomáš Zelenka , Alica Chroňáková and Kateřina Petříčková

## Slide 2
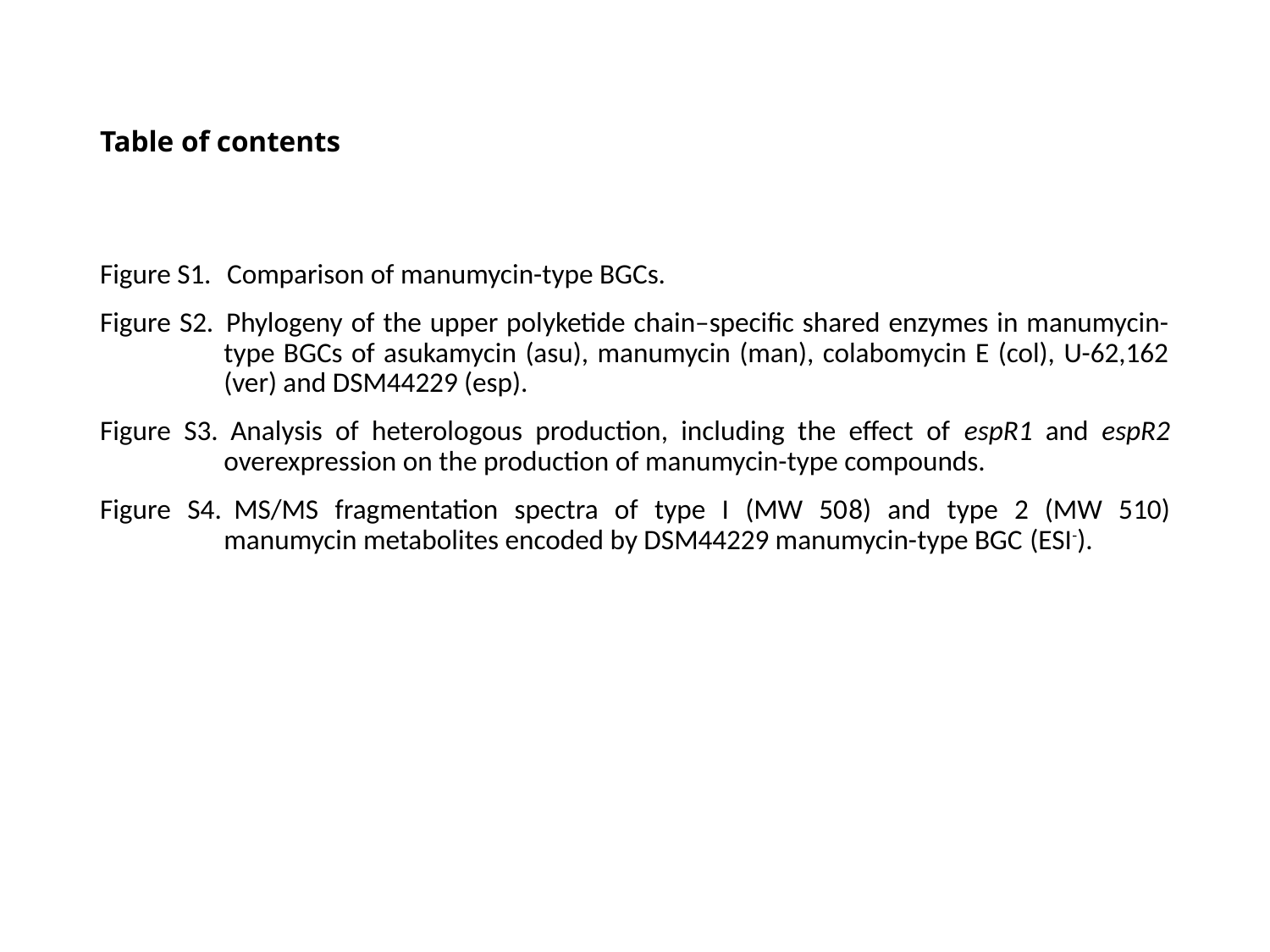

# Table of contents
Figure S1.	Comparison of manumycin-type BGCs.
Figure S2.	Phylogeny of the upper polyketide chain–specific shared enzymes in manumycin-type BGCs of asukamycin (asu), manumycin (man), colabomycin E (col), U-62,162 (ver) and DSM44229 (esp).
Figure S3.	Analysis of heterologous production, including the effect of espR1 and espR2 overexpression on the production of manumycin-type compounds.
Figure S4.	MS/MS fragmentation spectra of type I (MW 508) and type 2 (MW 510) manumycin metabolites encoded by DSM44229 manumycin-type BGC (ESI-).

## Slide 3
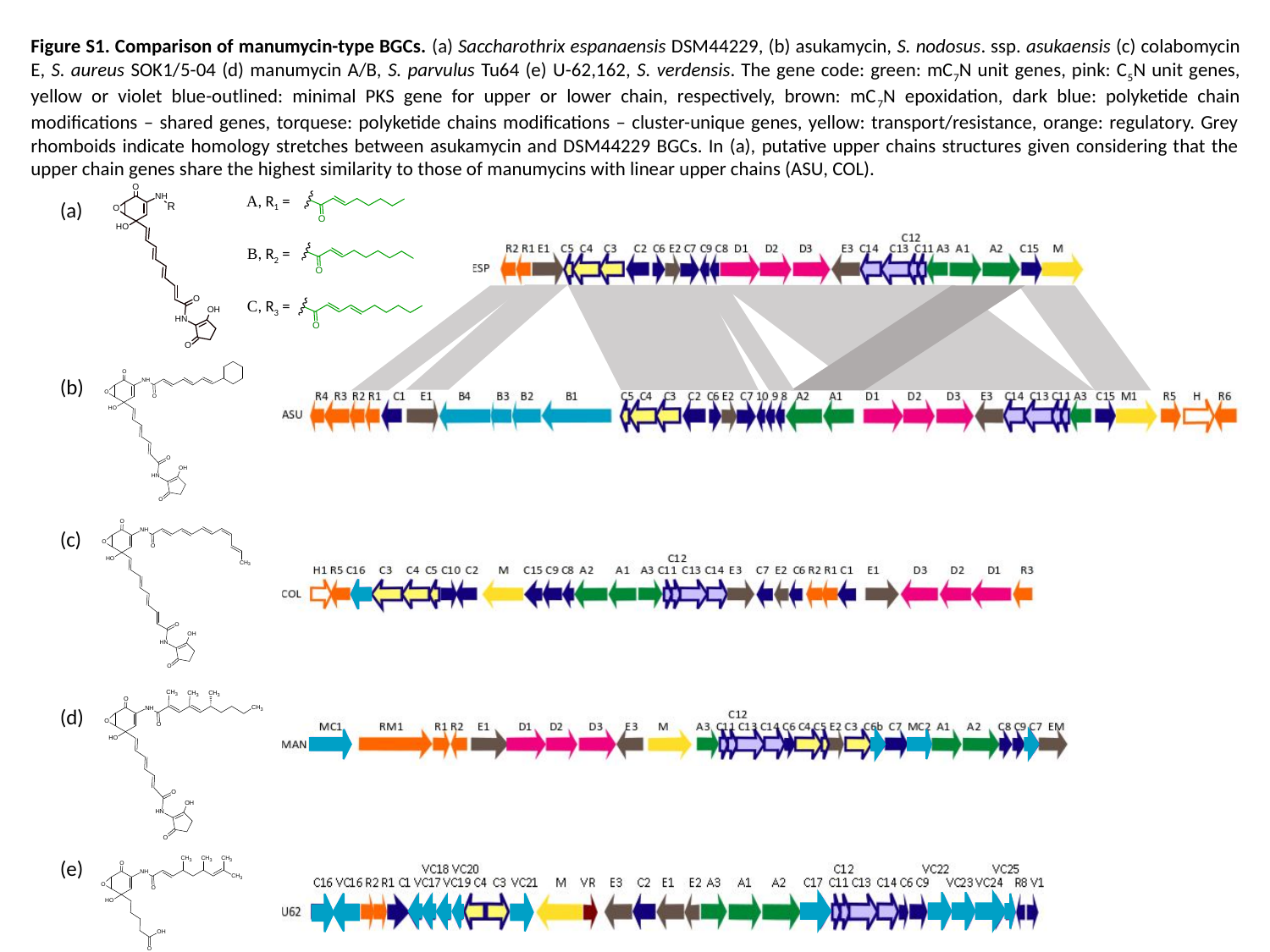

Figure S1. Comparison of manumycin-type BGCs. (a) Saccharothrix espanaensis DSM44229, (b) asukamycin, S. nodosus. ssp. asukaensis (c) colabomycin E, S. aureus SOK1/5-04 (d) manumycin A/B, S. parvulus Tu64 (e) U-62,162, S. verdensis. The gene code: green: mC7N unit genes, pink: C5N unit genes, yellow or violet blue-outlined: minimal PKS gene for upper or lower chain, respectively, brown: mC7N epoxidation, dark blue: polyketide chain modifications – shared genes, torquese: polyketide chains modifications – cluster-unique genes, yellow: transport/resistance, orange: regulatory. Grey rhomboids indicate homology stretches between asukamycin and DSM44229 BGCs. In (a), putative upper chains structures given considering that the upper chain genes share the highest similarity to those of manumycins with linear upper chains (ASU, COL).
A, R1 =
B, R2 =
C, R3 =
(a)
(b)
(c)
(d)
(e)

## Slide 4
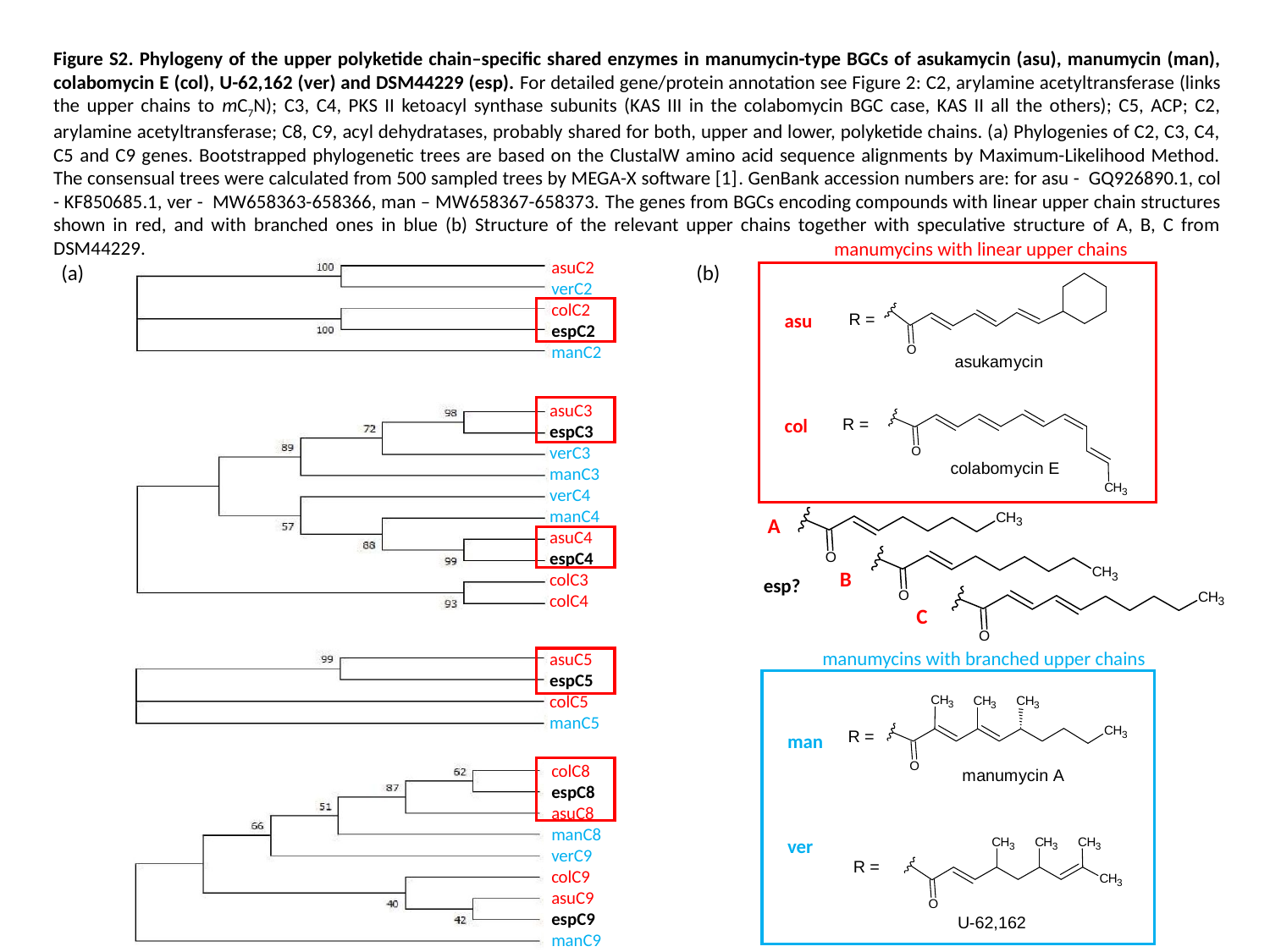

Figure S2. Phylogeny of the upper polyketide chain–specific shared enzymes in manumycin-type BGCs of asukamycin (asu), manumycin (man), colabomycin E (col), U-62,162 (ver) and DSM44229 (esp). For detailed gene/protein annotation see Figure 2: C2, arylamine acetyltransferase (links the upper chains to mC7N); C3, C4, PKS II ketoacyl synthase subunits (KAS III in the colabomycin BGC case, KAS II all the others); C5, ACP; C2, arylamine acetyltransferase; C8, C9, acyl dehydratases, probably shared for both, upper and lower, polyketide chains. (a) Phylogenies of C2, C3, C4, C5 and C9 genes. Bootstrapped phylogenetic trees are based on the ClustalW amino acid sequence alignments by Maximum-Likelihood Method. The consensual trees were calculated from 500 sampled trees by MEGA-X software [1]. GenBank accession numbers are: for asu - GQ926890.1, col - KF850685.1, ver - MW658363-658366, man – MW658367-658373. The genes from BGCs encoding compounds with linear upper chain structures shown in red, and with branched ones in blue (b) Structure of the relevant upper chains together with speculative structure of A, B, C from DSM44229.
manumycins with linear upper chains
asuC2
verC2
colC2
espC2
manC2
(a)					(b)
asu
col
asuC3
espC3
verC3
manC3
verC4
manC4
asuC4
espC4
colC3
colC4
A
B
esp?
C
manumycins with branched upper chains
asuC5
espC5
colC5
manC5
man
ver
colC8
espC8
asuC8
manC8
verC9
colC9
asuC9
espC9
manC9

## Slide 5
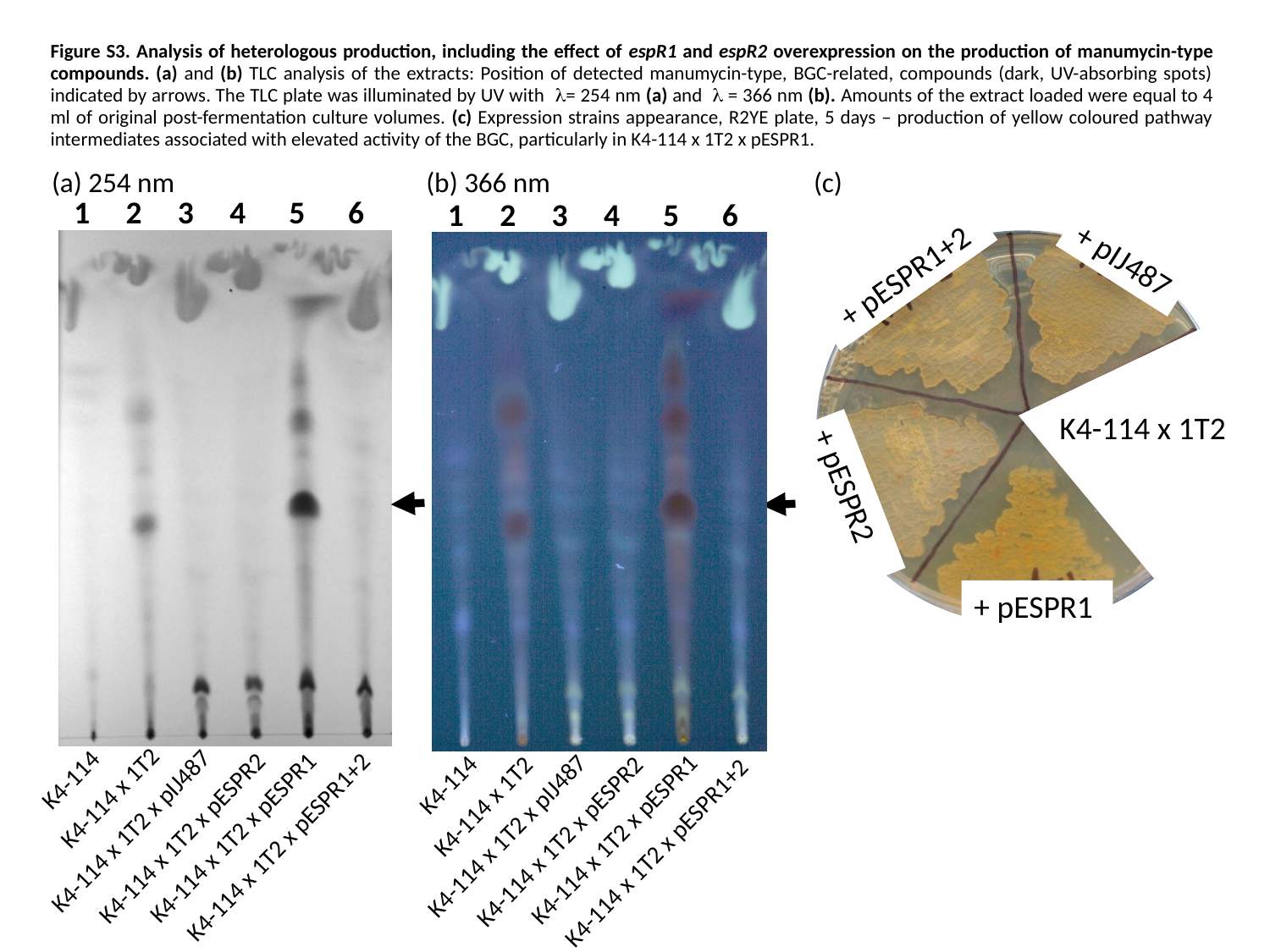

Figure S3. Analysis of heterologous production, including the effect of espR1 and espR2 overexpression on the production of manumycin-type compounds. (a) and (b) TLC analysis of the extracts: Position of detected manumycin-type, BGC-related, compounds (dark, UV-absorbing spots) indicated by arrows. The TLC plate was illuminated by UV with l= 254 nm (a) and l = 366 nm (b). Amounts of the extract loaded were equal to 4 ml of original post-fermentation culture volumes. (c) Expression strains appearance, R2YE plate, 5 days – production of yellow coloured pathway intermediates associated with elevated activity of the BGC, particularly in K4-114 x 1T2 x pESPR1.
(a) 254 nm	 (b) 366 nm			(c)
1 2 3 4 5 6
1 2 3 4 5 6
+ pIJ487
 + pESPR1+2
K4-114 x 1T2
+ pESPR2
+ pESPR1
K4-114
K4-114
K4-114 x 1T2
K4-114 x 1T2
K4-114 x 1T2 x pIJ487
K4-114 x 1T2 x pIJ487
K4-114 x 1T2 x pESPR1
K4-114 x 1T2 x pESPR2
K4-114 x 1T2 x pESPR1
K4-114 x 1T2 x pESPR2
K4-114 x 1T2 x pESPR1+2
K4-114 x 1T2 x pESPR1+2

## Slide 6
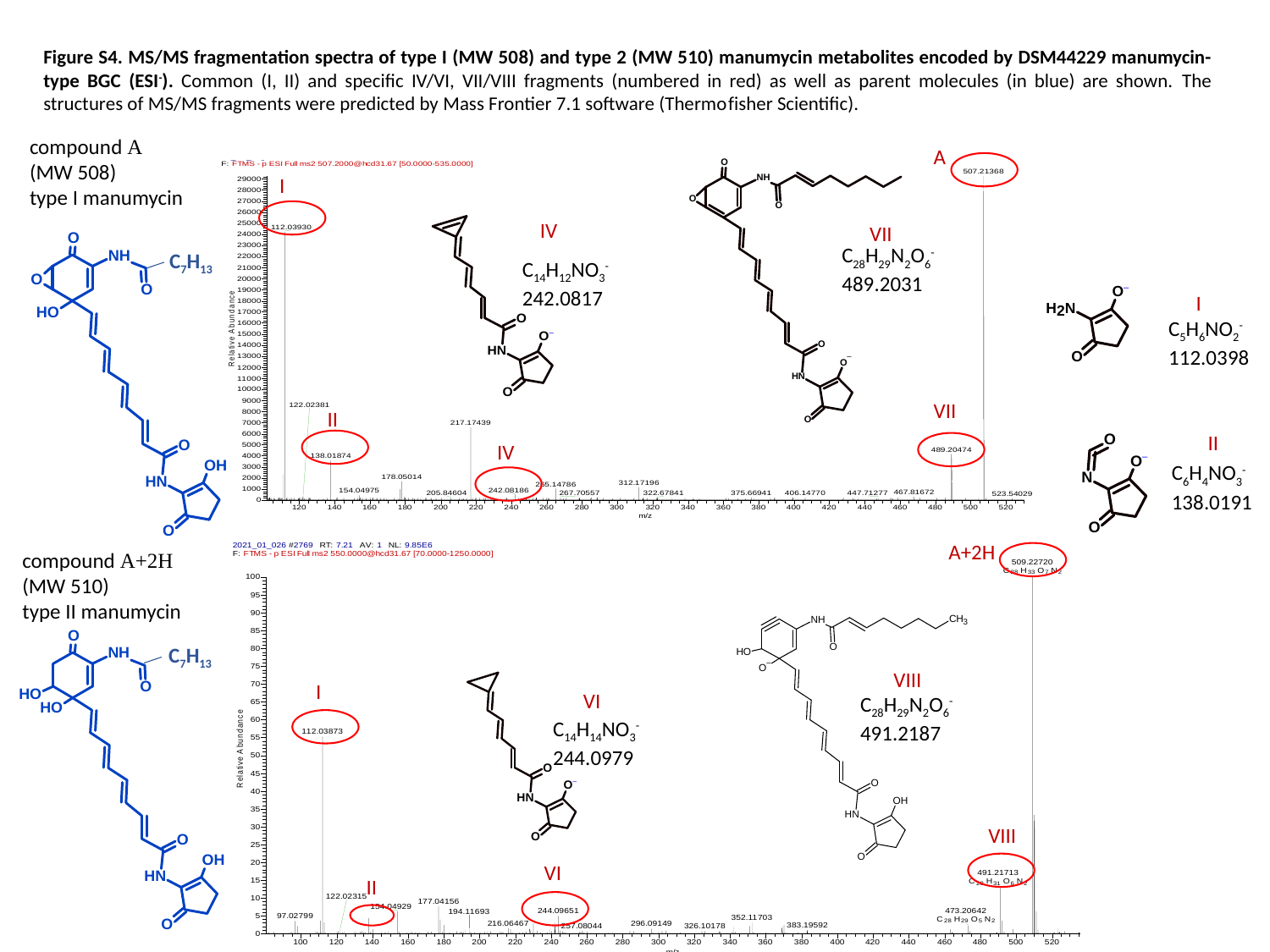

Figure S4. MS/MS fragmentation spectra of type I (MW 508) and type 2 (MW 510) manumycin metabolites encoded by DSM44229 manumycin-type BGC (ESI-). Common (I, II) and specific IV/VI, VII/VIII fragments (numbered in red) as well as parent molecules (in blue) are shown. The structures of MS/MS fragments were predicted by Mass Frontier 7.1 software (Thermofisher Scientific).
compound A
(MW 508)
type I manumycin
A
I
IV
VII
C7H13
C28H29N2O6-
489.2031
C14H12NO3-
242.0817
I
C5H6NO2-
112.0398
VII
II
II
IV
C6H4NO3-
138.0191
A+2H
compound A+2H
(MW 510)
type II manumycin
C7H13
VIII
I
VI
C28H29N2O6-
491.2187
C14H14NO3-
244.0979
VIII
VI
II

## Slide 7
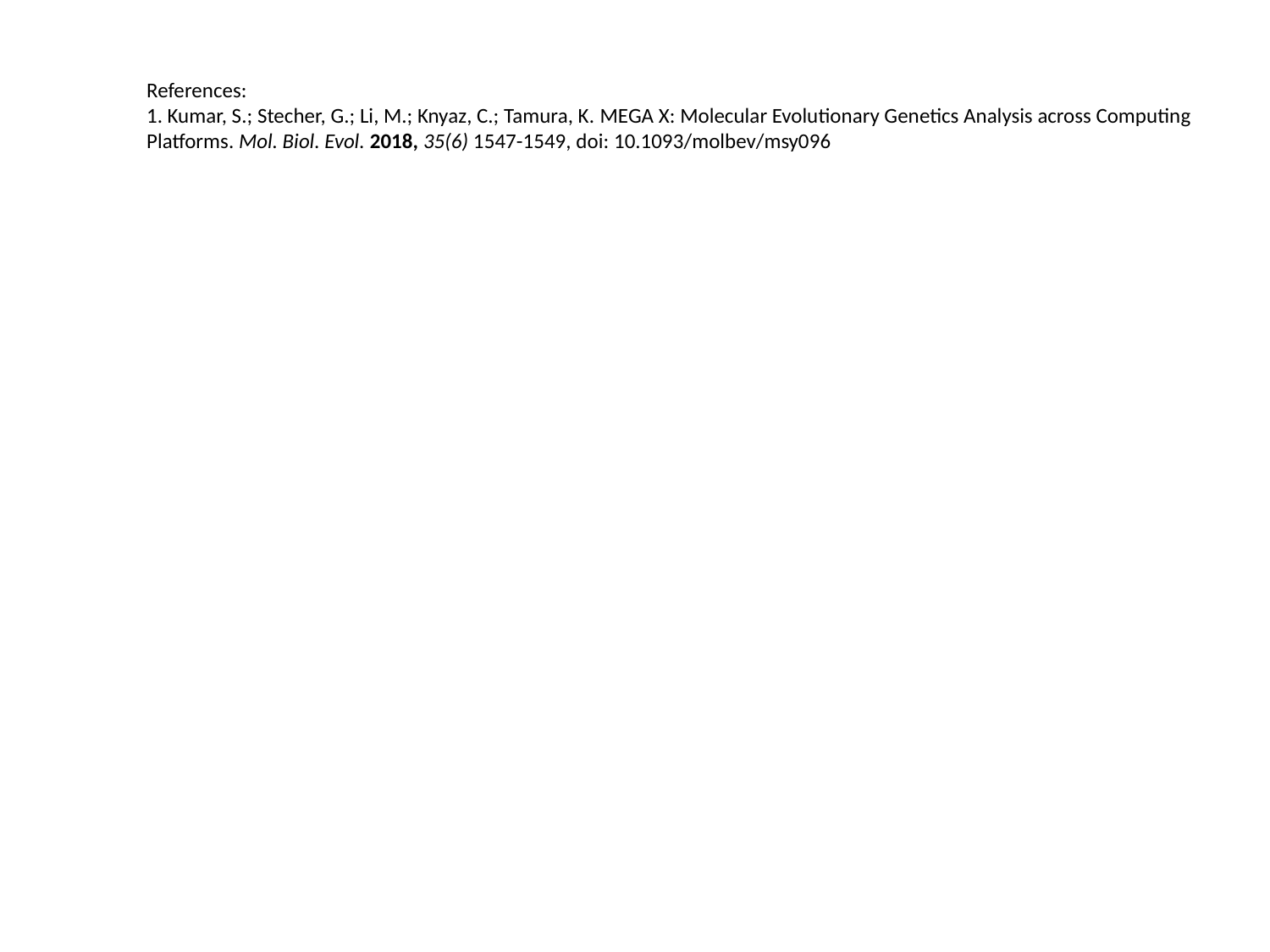

References:
1. Kumar, S.; Stecher, G.; Li, M.; Knyaz, C.; Tamura, K. MEGA X: Molecular Evolutionary Genetics Analysis across Computing Platforms. Mol. Biol. Evol. 2018, 35(6) 1547-1549, doi: 10.1093/molbev/msy096
